# Racial differences in laboratory testing as a potential mechanism for bias in AI: A matched cohort analysis in emergency department visits

**DOI:** 10.1371/journal.pgph.0003555

**Published:** 2024-10-30

**Authors:** Trenton Chang, Mark Nuppnau, Ying He, Keith E. Kocher, Thomas S. Valley, Michael W. Sjoding, Jenna Wiens

**Affiliations:** 1 Division of Computer Science and Engineering, University of Michigan, Ann Arbor, Michigan, United States of America; 2 Division of Pulmonary and Critical Care, Michigan Medicine, University of Michigan, Ann Arbor, Michigan, United States of America; 3 VA Center for Clinical Management Research, Ann Arbor, Michigan, United States of America; 4 Departments of Emergency Medicine and Learning Health Sciences, Michigan Medicine, University of Michigan, Ann Arbor, Michigan, United States of America; University of Global Health Equity, RWANDA

## Abstract

AI models are often trained using available laboratory test results. Racial differences in laboratory testing may bias AI models for clinical decision support, amplifying existing inequities. This study aims to measure the extent of racial differences in laboratory testing in adult emergency department (ED) visits. We conducted a retrospective 1:1 exact-matched cohort study of Black and White adult patients seen in the ED, matching on age, biological sex, chief complaint, and ED triage score, using ED visits at two U.S. teaching hospitals: Michigan Medicine, Ann Arbor, MI (U-M, 2015–2022), and Beth Israel Deaconess Medical Center, Boston, MA (BIDMC, 2011–2019). Post-matching, White patients had significantly higher testing rates than Black patients for complete blood count (BIDMC difference: 1.7%, 95% CI: 1.1% to 2.4%, U-M difference: 2.0%, 95% CI: 1.6% to 2.5%), metabolic panel (BIDMC: 1.5%, 95% CI: 0.9% to 2.1%, U-M: 1.9%, 95% CI: 1.4% to 2.4%), and blood culture (BIDMC: 0.9%, 95% CI: 0.5% to 1.2%, U-M: 0.7%, 95% CI: 0.4% to 1.1%). Black patients had significantly higher testing rates for troponin than White patients (BIDMC: -2.1%, 95% CI: -2.6% to -1.6%, U-M: -2.2%, 95% CI: -2.7% to -1.8%). The observed racial testing differences may impact AI models trained using available laboratory results. The findings also motivate further study of how such differences arise and how to mitigate potential impacts on AI models.

## Introduction

Racial differences in the delivery of clinical care disproportionately harm racial minorities. For example, multiple studies have found lower rates of colon cancer screenings and increased all-cause mortality among Black patients [1]. AI models could amplify biases present in the data, further hindering health equity. In particular, AI models may rely on spurious correlations or confounded relationships to predict outcomes of interest [2,3]. For example, AI models trained on data that reflect racial health disparities (*e*.*g*., differences in healthcare spending) under-recommend Black patients for high-risk care management programs [4]. Without accounting for spurious correlations between race and the clinical outcome of interest, AI models may encode and amplify racial inequities in the delivery of clinical care.

Racial differences in laboratory testing rates are a potentially widespread source of bias in AI models for healthcare settings. Many AI models are developed to predict clinical outcomes in which the pragmatic definition depends on laboratory test results (e.g., sepsis [5,6]), such that untested patients are assumed to have a normal result. This assumption is often operationalized by assigning untested patients a negative label during model training [7–12]. However, under this assumption, if laboratory testing rates differ across race, AI models may exhibit disparate performance across racial subgroups [13]. Such models could inappropriately underestimate risk for patients in racial groups less likely to receive laboratory tests, potentially amplifying inequities in clinical care. While previous work [14,15] has examined racial differences in specific laboratory tests in patient subpopulations, to the best of our knowledge, a large-scale study of racial differences in laboratory testing has not been performed.

We performed a retrospective cohort study of laboratory testing differences between Black and White patients visiting the emergency department (ED) at two large U.S. teaching hospitals. We analyzed the ED since it features a wide range of populations and clinical conditions with ample diagnostic data. Furthermore, patients are not pre-selected for care and generally do not choose their providers. ED patient cohorts are also commonly used in training AI models for predicting clinical outcomes [7,9,16–18]. We quantified testing rates within Black and White patients across seven common laboratory tests with and without matching on age, biological sex, chief complaint, and ED triage score.

## Methods

### Study population

The two study cohorts included all patients seen in the emergency department (ED) between 2011–2019 at Beth Israel Deaconess Medical Center (BIDMC), Boston, MA via the publicly-available MIMIC-IV dataset (Medical Information Mart for Intensive Care, version 2.0), and between 2015–2022 at Michigan Medicine (U-M), in Ann Arbor, MI [19]. Both institutions are teaching hospitals, and both datasets comprise electronic health records that include demographic and clinical data collected during hospital visit(s), with patient and ED visit identifiers for linking data tables. MIMIC-IV is also one of the most widely used clinical datasets for AI model training. We restrict the analysis to White and Black patients, the two most common racial groups at each institution. We exclude psychiatric admissions due to low rates of laboratory testing. Data for the BIDMC was downloaded as authorized by the data usage agreement on August 15, 2022, and accessed for this study between August 15, 2022, and December 8, 2023. Data for the U-M cohort was accessed for this study between April 13, 2023, and September 21, 2023.

### Extracting patient and ED visit characteristics

We collected race, age, biological sex, free-text chief complaint, and ED triage scores from the electronic health record based on information gathered in each ED visit. Race was provided as a text string based on information collected during patient registration. At BIDMC, race labels sometimes included nationality. Thus, we defined patients as White if their race was of the form “WHITE” or “WHITE–[nationality],” and likewise for Black patients. Furthermore, BIDMC (but not U-M) records “HISPANIC/LATINO” as a distinct racial category. Thus, we were unable to include White and Black Hispanic/Latino patients in our cohort. At U-M, patients were defined as White if their race was “Caucasian,” and Black if their race was “African American.” For both institutions, patients with unknown/missing race were excluded from the analysis. At BIDMC and U-M, biological sex was provided via fields called “Gender” and “GenderName,” respectively. Since very few records contained values other than “Male” or “Female” (BIDMC: 0 [0.0%], U-M: 14 [0.0%]), we interpreted this variable as biological sex.

To extract chief complaint information, the free-text chief complaint was case-normalized and sorted at the word level to merge duplicate complaints (*e*.*g*., “abdominal pain, chest pain” vs. “chest pain, abdominal pain”). ED triage score is an integer from 1 (most urgent) to 5 (least urgent), representing a patient’s medical needs, typically assigned by an ED triage nurse at intake using pre-specified, structured criteria. ED triage score represents the priority for which ED patients should be treated and is a proxy for illness severity.

### Outcome measure

We measured laboratory testing rates for the following common laboratory tests in Black and White patients: complete blood count (CBC) with and without differential, basic or comprehensive metabolic panel, arterial blood gas (ABG), blood cultures, troponin T, brain natriuretic peptide (BNP), and d-dimer.

### Statistical analysis

Prior to matching, we compared demographics across White versus Black patients within ED visits in each cohort. We measured the distributions of biological sex (percent female), hospital admission rate (including observational status), the median and interquartile range of age, the ED triage score distribution, and laboratory testing rates. Laboratory testing rate was defined as the percentage of ED visits in which a test was administered.

We then performed a 1:1 exact match across White and Black patients on age, biological sex, chief complaint, and ED triage score at the ED visit level within each site to control for variables that potentially explain racial differences in testing rate. We limited the set of matching variables since some candidate matching variables could encode systematic biases (*e*.*g*., healthcare utilization). Individuals with no match were excluded. Since ED triage score could be assessed differently in Black versus White patients for the same medical problem [20,21], we repeated the matched analysis without ED triage score as a sensitivity analysis. Within matched cohorts, we compared laboratory testing rates between White and Black patients and computed testing differences disaggregated by biological sex and admission status (whether an ED visit resulted in discharge or admission to the hospital).

For hypothesis testing, we used two-sided two-sample *z*-tests for proportions [22] for differences in rates across race, the Mann-Whitney U-test [23] for differences in age, and the chi-squared test for independence [24] for ED triage score (df = 5), treating unknown missing or triage scores as a separate category. We also report the mean and standard deviation for ED triage scores within White and Black patients (no hypothesis tested), since the chi-squared test is not directional. We assessed statistical significance at a 5% level and applied a Bonferroni correction for multiple hypotheses [25] for the primary analysis (N = 44). In secondary analyses, we used correction factors N = 14 for the subgroup analyses and N = 28 for the analysis disaggregated by biological sex. No correction is applied for the sensitivity analysis since it is exploratory. We also compute parametric 95% confidence intervals for all differences in proportions [22]. Full methodological details are provided in S1 Appendix.

### Ethics statement

This analysis was deemed exempt by the U-M IRB (IRBMED) under Exemption 4(iii) at 45 CFR 46.104(d) in the U.S. Code of Federal Regulations, which is applied for secondary research for which consent is not required. Research involving only information collection and analysis as in our study is covered under Exemption 4(iii) of the relevant statute. The exemption included a Waiver of HIPAA Authorization. This study was determined by the IRB to conform with applicable regulations and policies.

## Results

**Fig 1:**
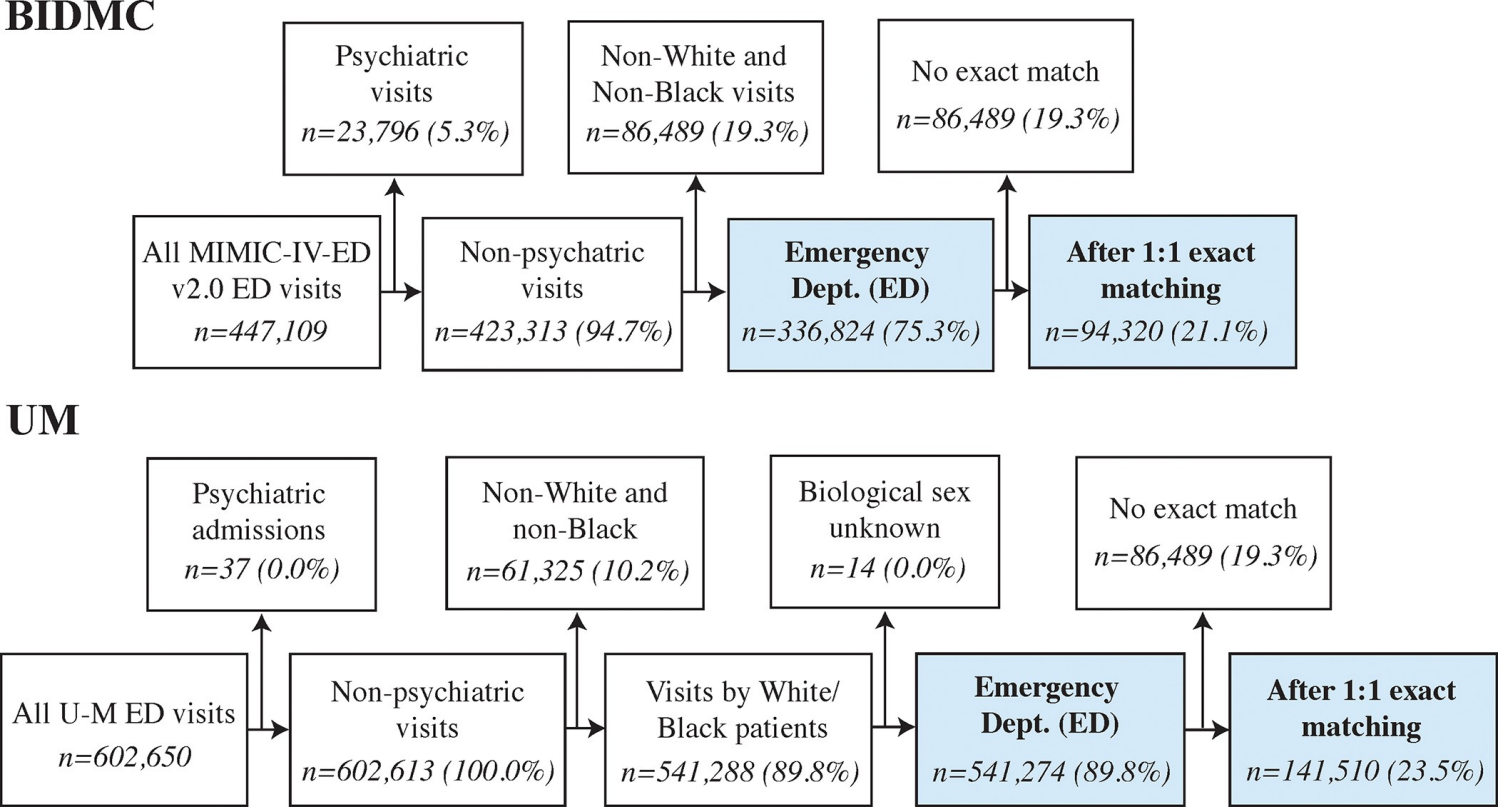
Cohort diagrams for BIDMC and U-M. Cohort diagram of patient groups selected for study at BIDMC (top) and U-M (bottom). Note that at BIDMC, there were no ED visits in BIDMC associated with individuals of unknown sex. Numbers may not sum to 100.0% due to rounding.

After applying exclusion criteria (Fig 1), we analyzed 336,824 and 541,310 ED visits at BIDMC and U-M, respectively (Table 1). At BIDMC and U-M, Black patients accounted for 92,437 (27.4%) and 102,313 (18.9%) ED visits, respectively. In the unmatched cohort, White patients were significantly older than Black patients (BIDMC: 55 vs. 46 years, difference: 9 years, p < .001; U-M: 52 vs. 43 years, difference: 9 years, p < .001) and were less often female (BIDMC: 52.0% vs. 62.0%, 95% CI: -10.5% to -9.3, p < .001, U-M: 53.1% vs. 57.0%, 95% CI: -4.5% to -3.5%, p < .001). The ED triage score distribution also differed significantly across White and Black patients (p < .001). On average, White patients were assessed as more ill than Black patients (*i*.*e*., lower ED triage score; BIDMC: 2.6 vs. 2.8, difference: -0.2; U-M: 2.7 vs. 2.8, difference: -0.1). At BIDMC and U-M, 157,520 (46.8%) and 196,918 (36.4%) patients were admitted to the hospital following the ED visit, respectively. White patients were admitted at significantly higher rates than Black patients (BIDMC: 51.0% vs. 36.0%, difference 15.0%, 95% CI: 14.3% to 15.5%, p < .001; U-M: 37.9% vs. 29.8%, difference 8.1%, 95% CI: 7.6% to 8.6%, p < .001). We also report the 25 most common chief complaints by race at each institution (S1 Table).

**Table 1:** Cohort demographics for BIDMC (left) and U-M (right) among all ED visits for the unmatched cohort.

Institution	BIDMC			U-M		
Race	White (n = 244,387)	Black (n = 92,437)	*P* value	White (n = 438,997)	Black (n = 102,313)	*P* value
Median age, years (IQR^a^)	55 (35–71)	46 (30–61)	< .001	52 (34–67)	43 (30–58)	< .001
Sex (% Female)^b^	127,213 (52.0)	57,271 (62.0)	< .001	233,022 (53.1)	58,344 (57.0)	< .001
ED triage score (%)^c^						
1	15,271 (6.3)	3,847 (4.2)	< .001	5,630 (1.3)	796 (0.8)	< .001
2	86,063 (35.2)	24,021 (26.0)		177,355 (40.4)	35,016 (34.2)	
3	126,126 (51.6)	54,620 (59.1)		202,633 (46.1)	49,977 (48.9)	
4	13,106 (5.4)	8,796 (9.5)		38,815 (8.8)	13,123 (12.8)	
5	520 (0.2)	325 (0.4)		3,659 (0.8)	1,018 (1.0)	
Missing	3,301 (1.4)	828 (0.9)		10,913 (2.5)	2,339 (2.3)	
Mean ED triage score (SD)^d^	2.57 (0.70)	2.76 (0.69)	N/A	2.67 (0.69)	2.79 (0.71)	N/A
Admitted to hospital (%)	124,310 (50.9)	33,210 (35.9)	< .001	166,405 (37.9)	30,513 (29.8)	< .001

^a^Interquartile range.

^b^Male vs. female were the only two options in the MIMIC-IV dataset.

^c^Percentages may not sum to 100.0% due to rounding.

^d^Missing ED triage scores excluded.

**Fig 2:**
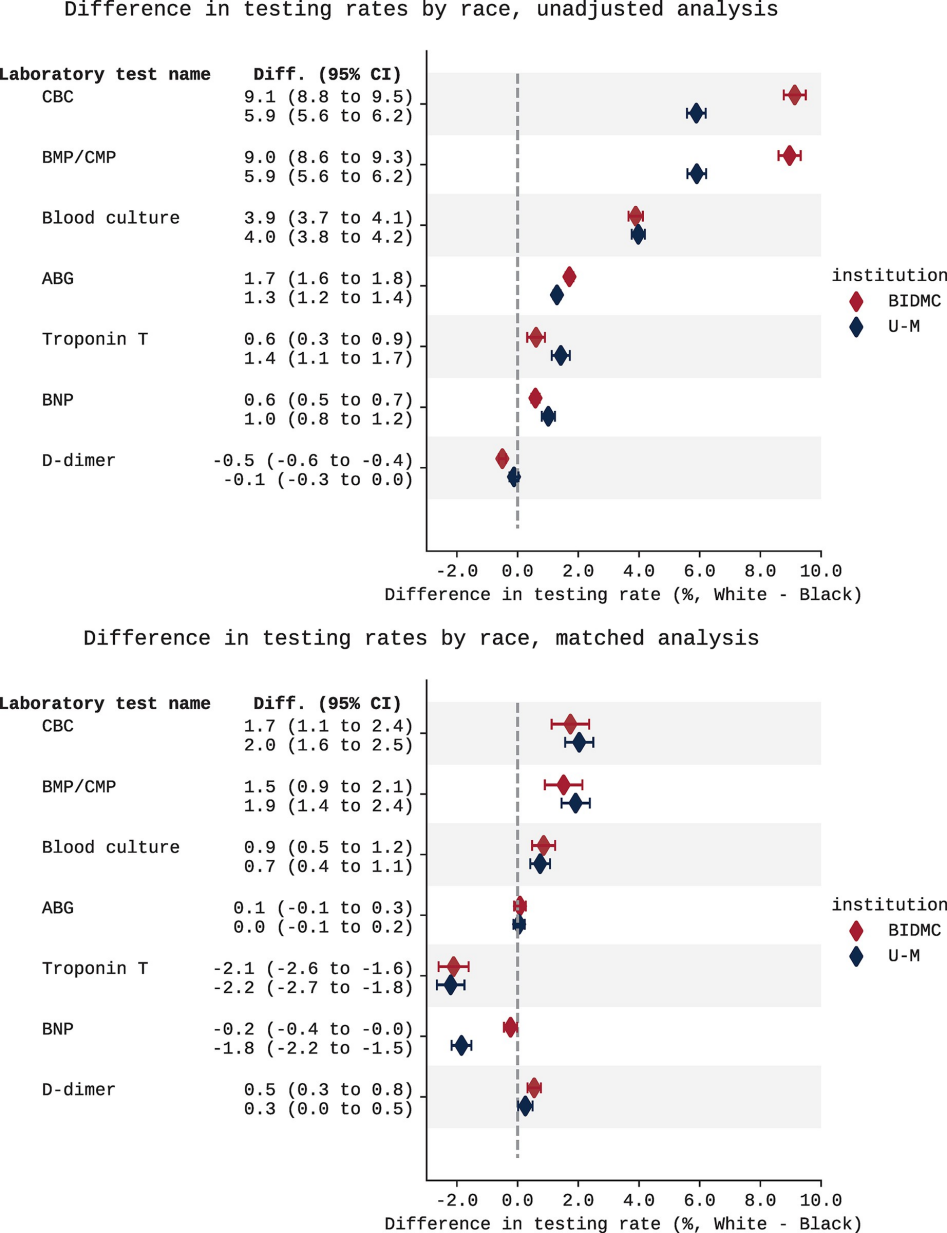
Differences in testing rates between White and Black patients, with and without matching on age, sex, chief complaint, and ED triage score. Forest plots show difference in testing rate across White vs. Black patients (White %—Black %), with no matching adjustment (top), and matched on age, sex, chief complaint, and ED triage score (bottom). Differences at BIDMC are shown in red; differences at U-M are shown in blue. Actual values shown in S2 Table (results with no matching adjustment) and S4 Table (matched analysis).

The unmatched analysis also found that White patients were significantly more likely than Black patients to receive all laboratory tests in our analysis except d*-*dimer (Fig 2, top). For example, White patients were significantly more likely than Black patients to receive a CBC (BIDMC: 69.6% vs. 60.5%, difference 9.1%, 95% CI: 8.5% to 9.8%, p < .001). Fig 2 (top) shows similar findings for metabolic panels, blood cultures, ABG, troponin tests, and BNP (*i*.*e*., all other tests analyzed other than d-dimer). Trends were similar at U-M. See S2 Table for the testing rates shown in Fig 2 (top).

**Fig 3:**
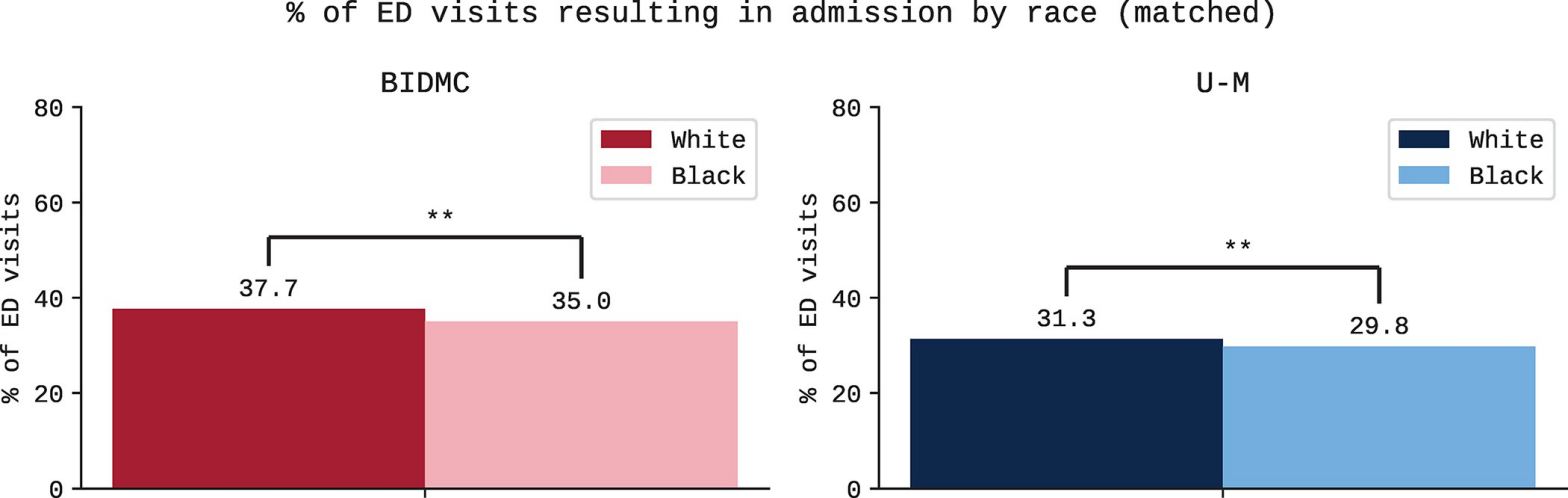
Differences in admission rates between White and Black patients matched on age, sex, chief complaint, and ED triage score. Bar plots show difference in rate of admission to the hospital among ED visits in study cohort grouped by site. Differences at BIDMC are shown in red; differences at U-M are shown in blue. “**” denotes statistical significance at 5% level. Actual values are shown in S3 Table.

Exact 1:1 matching on age, sex, ED triage score, and chief complaint yielded 94,320 and 141,510 ED visits at BIDMC and U-M, respectively. At BIDMC, this cohort included 51.0% and 19.3% of ED visits by Black and White patients, respectively. At U-M, this cohort included 69.2% and 16.1% of ED visits by Black and White patients, respectively. Matched cohort demographics can be found in S3 Table. Matching narrowed differences in testing rates (Fig 2). White patients remained significantly more likely than Black patients to receive a CBC (BIDMC: 63.0% vs. 61.3%, difference 1.7%, 95% CI: 1.1% to 2.4%, p < .001), metabolic panel (BIDMC: 63.2% vs. 61.7%, difference 1.5%, 95% CI: 0.9% to 2.1%, p < .001), or blood culture (BIDMC: 10.3% vs. 9.5%, difference 0.9%, 95% CI: 0.5% to 1.2%, p < .001), while White patients were significantly less likely than Black patients to receive a troponin test (BIDMC: 17.2% vs. 19.3%, difference -2.1%, 95% CI: -2.6% to -1.6%, p < .001). Differences in laboratory testing were not statistically significant at both institutions for BNP, *d*-dimer, or ABG. Trends were similar at U-M (Fig 2, bottom). See S4 Table for the testing rates shown in Fig 2 (bottom). White patients remained significantly more likely than Black patients to be admitted to the hospital after an ED visit at both institutions (BIDMC: 37.7% vs. 35.0%, difference 2.7%, 95% CI: 2.0% to 3.3%, p < .001; Fig 3, left). Trends were similar at U-M (Fig 3, right).

As a sensitivity analysis, we repeated the matched analysis without ED triage scores. Results were similar. White patients were significantly more likely than Black patients to receive a CBC, metabolic panel, and blood culture, and significantly less likely than Black patients to receive a troponin test (S1 Fig and S5 Table). White patients also remained more likely to be admitted to the hospital (S2 Fig). The analysis disaggregated by biological sex found similar trends: within male and female patients, White patients were significantly more likely than Black patients to receive a CBC or metabolic panel, and significantly less likely than Black patients to receive a troponin test (S3 Fig and S6 Table). Disaggregating by admission status narrowed some testing differences in visits resulting in discharge (S4 Fig and S7 Table) and visits resulting in admission (S4 Fig and S8 Table). However, given the significant differences in admission rate across race, narrower testing differences do not necessarily signal less systematic bias.

## Discussion

Before presenting our findings, as an exercise in reflexivity, we as authors acknowledge how our positionality influenced this project. A subset of the authors serve as clinicians in the ED and the ICU at one of the hospitals in our analyses. Another subset of our authors are machine learning researchers. Several of the authors have prior work on health disparities research. In addition, one author had clinical training at a hospital that cared for a predominantly Black patient population. The authors identify as a White North American woman, White North American men, Asian Indian American man, Asian Taiwanese American man, and a female first-generation immigrant to the United States from Asia. None of the authors have lived experiences with anti-Black racism. It was our prior research in addition to the education received around implicit bias and racial disparities in access to clinical care that inspired, in part, the initial hypothesis.

We acknowledge that, as an observational study, our work has some extractive elements, since the authors do not interact with the communities from which data are sourced. Furthermore, our work is unlikely to immediately and directly remediate inequities in the delivery of clinical care, despite our alignment with equitable goals. However, using the power of our positionality as computer scientists and clinicians at a research institution, we aim to amplify cautionary messages about AI with respect to marginalized populations.

We now summarize the findings. Across six of seven laboratory tests, in the unmatched cohort, Black patients had significantly lower testing rates than White patients. After matching to account for differences in clinical characteristics, Black patients remained significantly less likely to receive a CBC or metabolic panel, while White patients were significantly less likely to receive a troponin or BNP test. Hospital admission partially explained racial differences in laboratory testing rates, with Black patients significantly less likely to be admitted to the hospital. Given similar findings across two institutions, these trends may hold more broadly.

Although this study is unable to examine the reasons for laboratory test orders, testing differences could impact AI models trained to predict outcomes defined using laboratory test results. For example, Black patients were found to be significantly less likely to receive blood cultures. They may subsequently be less likely to be diagnosed with serious infections. AI models trained to predict this outcome may underestimate risk in Black patients since models are generally trained to optimize a performance metric (*e*.*g*., accuracy) without explicitly accounting for confounding (*i*.*e*., between race and the outcome of interest). Specifically, such models could ultimately “learn” that Black patients are less likely to experience serious infections without “accounting” for lower admission rates and lower testing probability.

While quantifying differences in AI model performance across race under biased laboratory testing is not possible without “ground truth” diagnosis labels, studies in similar problem settings have consistently demonstrated the potential for harm. Past work has documented disparate performance in AI models with respect to race in healthcare applications [4,26]. Furthermore, a simulation study of disparities in testing across population subgroups suggests ample potential for performance gaps to emerge under biased laboratory testing patterns [13], such as those found in this study. Ultimately, acting on predictions of AI models trained on data exhibiting racial differences in laboratory testing could reinforce undertesting in marginalized patient populations. Furthermore, since laboratory tests are often used as a source of signal for a variety of downstream diagnoses, even these seemingly small differences could have widespread impacts on the equitable delivery of clinical care.

In general, machine learning researchers do not adjust for confounding prior to training ML models. Potential solutions for addressing selection bias are well-studied but may have limited practical utility for correcting biases in laboratory testing. For example, in the econometric and epidemiology literature, methods such as reweighting or matching are standard in observational studies to adjust for selection bias/survey sampling bias [27,28]. These approaches are most useful when there is no unobserved confounding, which is difficult to guarantee in practice. One may also consider removing race as an input to the model, but such approaches are insufficient if other variables are correlated with race (*i*.*e*., proxy discrimination [29,30]). In certain situations, it may be appropriate to train AI models on a sub-cohort where the spurious correlation is weaker (*e*.*g*., admitted or tested patients). However, this technique may discard most available training data, leading to lower model performance. Furthermore, the resulting model would not apply beyond the sub-cohort used for training (*e*.*g*., predicting serious infections in patients without a blood culture, using data from patients who received a blood culture) [17,31].

Thus, we require novel approaches to mitigate the potential impacts of racial testing differences on AI models. We highlight causally-motivated methods as a promising direction, since they could be used to model mechanisms of bias in laboratory testing and facilitate “counterfactual fairness” analyses (*e*.*g*., would a patient with these characteristics be tested regardless of their race) [32]. One may also consider extending techniques from semi-supervised/weakly-supervised learning (*i*.*e*., treating untested patients as unlabeled instead of negative), many of which assume that label missingness occurs randomly, motivating future methods that account for potentially biased laboratory testing patterns [33]. In parallel, it is imperative that we examine how racial testing differences emerge, to inform policies that minimize biases in laboratory testing, reducing the prevalence of spurious correlations in clinical data. Ultimately, due to the potential for AI to amplify existing harms, one should carefully consider how to adjust for racial differences in laboratory testing in AI models. A “color-blind” approach that ignores correlations between race and testing rates is unlikely to mitigate harm [34].

## Limitations

Our study has several limitations. First, race is not an objective/unambiguous construct, which is reflected in our data. Some racial categories in BIDMC could overlap with patients who identify as either White or Black (e.g., “Brazilian,” “Hispanic/Latino”) or mixed-race individuals. We excluded such patients from our study. Our definition of “White” and “Black” captures the majority (79.6%) of all ED visits in BIDMC, mitigating the impact of such ambiguity. There may also be heterogeneity in testing differences within White and Black patients, which this study is unable to capture [35]. Second, to simplify the analysis, we restricted the study to White and Black patients. We recognize that there may be testing differences across other racial and ethnic groups. Past work highlights the disproportionate burden of racial disparities in clinical care on Hispanic/Latino, American Indian/Alaska Native, and Native Hawaiian/Pacific Islander populations in the U.S [36].

Third, matching on additional variables (*e*.*g*., comorbidities, additional markers of illness severity, patient history) could further explain correlations between race and testing rates. Such variables may also be subject to bias. Furthermore, exact-matching on additional variables could induce statistical bias if many units are dropped, while relaxations to exact matching may yield incomparable matched cohorts [37]. Fourth, our cohorts are drawn from U.S. academic teaching hospitals, which may not accurately reflect standard care across the U.S. Fifth, this study was unable to determine the appropriateness of testing rates. Although some of our findings align with previous work on the disproportionate impact of undertreatment/underdiagnosis on Black patients in various clinical contexts [1,15,38], the differences we observed could represent either under- or over-testing. Further study of racial differences in testing is warranted. Lastly, due to differences in data format, the laboratory test extraction pipelines necessarily differed across institutions. This could result in over/undercounting of tests. These limitations have minor impacts since our main outcome measure is testing differences rather than testing rates.

## Conclusion

Black patients were observed to be significantly less likely than White patients to receive multiple laboratory tests across two large academic hospitals. Racial differences in testing narrowed or reversed after matching to account for differences in clinical characteristics. Hospital admission also partially explained these gaps. However, many racial testing differences remained significant. The findings highlight a need to better understand how racial differences in ED laboratory testing emerge, since the patterns observed in this study could represent either over- or under-testing. Furthermore, racial testing differences may amplify bias in AI models, since such models may spuriously associate race with laboratory test outcomes. Standard methods for addressing confounding may be insufficient to mitigate spurious correlations.

## Supporting information

S1 Fig Sensitivity analysis of matching strategy showing differences in testing rates between White and Black patients matched on age, sex, and chief complaint only. (TIFF)

S2 Fig Differences in rate of admission to hospital between White and Black patients matched on age, sex, and chief complaint. (TIFF)

S3 Fig Differences in testing rates between White and Black patients matched on age, biological sex, chief complaint, and ED triage score, stratified by male (top) and female (bottom) patients.
(TIFF)

S4 Fig Subgroup analysis of testing differences by admission status (discharge vs. admission) between White and Black patients matched on age, sex, chief complaint, and ED triage score. (TIFF)

S1 Table Case-normalized free-text chief complaint for 25 most common chief complaints in the unmatched cohort at BIDMC (left) and U-M (right) among White and Black patients.
(PDF)

S2 Table Differences in testing rates between White and Black patients, no matching. (PDF)

S3 Table Cohort demographics for BIDMC (left) and U-M (right) among all ED visits after matching on age, biological sex, chief complaint, and ED triage score. (PDF)

S4 Table Full results for differences in testing rates between White and Black patients matched on age, sex, chief complaint, and ED triage score. (PDF)

S5 Table Differences in testing rates between White and Black patients matched on age, sex, and chief complaint, all patients. (PDF)

S6 Table Differences in testing rates between White and Black patients matched on age, sex, chief complaint, and ED triage score among all patients, by biological sex. (PDF)

S7 Table Difference in testing rates between White and Black patients in ED visits resulting in discharge, matched on age, sex, chief complaint, and ED triage score. (PDF)

S8 Table Difference in testing rates between White and Black patients in ED visits resulting in admission, matched on age, sex, chief complaint, and ED triage score. (PDF)

S1 Appendix Methods and results supplement. (DOCX)

## References

[1] Berry J, Bumpers K, Ogunlade V, Glover R, Davis S, Counts-Spriggs M, et al. Examining Racial Disparities in Colorectal Cancer Care. J Psychosoc Oncol. 2009;27: 59–83. doi: 10.1080/07347330802614840 19197679

[2] Geirhos R, Jacobsen J-H, Michaelis C, Zemel R, Brendel W, Bethge M, et al. Shortcut learning in deep neural networks. Nat Mach Intell. 2020;2: 665–673. doi: 10.1038/s42256-020-00257-z

[3] DeGrave AJ, Janizek JD, Lee S-I. AI for radiographic COVID-19 detection selects shortcuts over signal. Nat Mach Intell. 2021;3: 610–619. doi: 10.1038/s42256-021-00338-7

[4] Obermeyer Z, Powers B, Vogeli C, Mullainathan S. Dissecting racial bias in an algorithm used to manage the health of populations. Science. 2019;366: 447–453. doi: 10.1126/science.aax2342 31649194

[5] Rhee C, Klompas M. Sepsis trends: increasing incidence and decreasing mortality, or changing denominator? J Thorac Dis. 2020;12: S89–S100. doi: 10.21037/jtd.2019.12.51 32148931 PMC7024753

[6] Singer M, Deutschman CS, Seymour CW, Shankar-Hari M, Annane D, Bauer M, et al. The Third International Consensus Definitions for Sepsis and Septic Shock (Sepsis-3). JAMA. 2016;315: 801. doi: 10.1001/jama.2016.0287 26903338 PMC4968574

[7] Adams R, Henry KE, Sridharan A, Soleimani H, Zhan A, Rawat N, et al. Prospective, multi-site study of patient outcomes after implementation of the TREWS machine learning-based early warning system for sepsis. Nat Med. 2022;28: 1455–1460. doi: 10.1038/s41591-022-01894-0 35864252

[8] Henry KE, Hager DN, Pronovost PJ, Saria S. A targeted real-time early warning score (TREWScore) for septic shock. Sci Transl Med. 2015;7. doi: 10.1126/scitranslmed.aab3719 26246167

[9] Kamran F, Tang S, Otles E, McEvoy DS, Saleh SN, Gong J, et al. Early identification of patients admitted to hospital for covid-19 at risk of clinical deterioration: model development and multisite external validation study. BMJ. 2022; e068576. doi: 10.1136/bmj-2021-068576 35177406 PMC8850910

[10] Hartvigsen T, Sen C, Brownell S, Teeple E, Kong X, Rundensteiner E. Early Prediction of MRSA Infections using Electronic Health Records: Proceedings of the 11th International Joint Conference on Biomedical Engineering Systems and Technologies. Funchal, Madeira, Portugal: SCITEPRESS—Science and Technology Publications; 2018. pp. 156–167. doi: 10.5220/0006599601560167

[11] Jehi L, Ji X, Milinovich A, Erzurum S, Rubin BP, Gordon S, et al. Individualizing Risk Prediction for Positive Coronavirus Disease 2019 Testing. Chest. 2020;158: 1364–1375. doi: 10.1016/j.chest.2020.05.580 32533957 PMC7286244

[12] McDonald SA, Medford RJ, Basit MA, Diercks DB, Courtney DM. Derivation With Internal Validation of a Multivariable Predictive Model to Predict COVID‐19 Test Results in Emergency Department Patients. Jones AE, editor. Acad Emerg Med. 2021;28: 206–214. doi: 10.1111/acem.14182 33249683 PMC7753649

[13] Chang T, Sjoding MW, Wiens J. Disparate Censorship & Undertesting: A Source of Label Bias in Clinical Machine Learning. In: Lipton Z, Ranganath R, Sendak M, Sjoding M, Yeung S, editors. Proceedings of the 7th Machine Learning for Healthcare Conference. PMLR; 2022. pp. 343–390. Available: https://proceedings.mlr.press/v182/chang22a.html. PMC10162497 37152303

[14] Notardonato LD, Langerman SS, Zhou J, Calip GS, Chiu BC-H, Derman BA. Racial Disparities in the Diagnostic Evaluation of Multiple Myeloma. Blood. 2021;138: 4116–4116. doi: 10.1182/blood-2021-146910

[15] Payne NR, Puumala SE. Racial Disparities in Ordering Laboratory and Radiology Tests for Pediatric Patients in the Emergency Department: Pediatr Emerg Care. 2013;29: 598–606. doi: 10.1097/PEC.0b013e31828e6489 23603649

[16] Taylor RA, Moore CL, Cheung K-H, Brandt C. Predicting urinary tract infections in the emergency department with machine learning. Dong Q, editor. PLOS ONE. 2018;13: e0194085. doi: 10.1371/journal.pone.0194085 29513742 PMC5841824

[17] Hong WS, Haimovich AD, Taylor RA. Predicting hospital admission at emergency department triage using machine learning. Dong Q, editor. PLOS ONE. 2018;13: e0201016. doi: 10.1371/journal.pone.0201016 30028888 PMC6054406

[18] Taylor RA, Pare JR, Venkatesh AK, Mowafi H, Melnick ER, Fleischman W, et al. Prediction of In‐hospital Mortality in Emergency Department Patients With Sepsis: A Local Big Data–Driven, Machine Learning Approach. Jones A, editor. Acad Emerg Med. 2016;23: 269–278. doi: 10.1111/acem.12876 26679719 PMC5884101

[19] Johnson A, Bulgarelli L, Pollard T, Horng S, Celi LA, Mark R. MIMIC-IV. PhysioNet; doi: 10.13026/6MM1-EK67

[20] Schrader CD, Lewis LM. Racial Disparity in Emergency Department Triage. J Emerg Med. 2013;44: 511–518. doi: 10.1016/j.jemermed.2012.05.010 22818646

[21] Shanmugam D, Pierson E. Quantifying Inequality in Underreported Medical Conditions. ArXiv211004133 Cs. 2021 [cited 8 Nov 2021]. Available: http://arxiv.org/abs/2110.04133.

[22] Fisher RA. Statistical methods for research workers. Breakthroughs in statistics: Methodology and distribution. Springer; 1970. pp. 66–70.

[23] Mann HB, Whitney DR. On a Test of Whether one of Two Random Variables is Stochastically Larger than the Other. Ann Math Stat. 1947;18: 50–60. doi: 10.1214/aoms/1177730491

[24] Pearson K. On the criterion that a given system of deviations from the probable in the case of a correlated system of variables is such that it can be reasonably supposed to have arisen from random sampling. Lond Edinb Dublin Philos Mag J Sci. 1900;50: 157–175. doi: 10.1080/14786440009463897

[25] Dunn OJ. Multiple Comparisons among Means. J Am Stat Assoc. 1961;56: 52–64. doi: 10.1080/01621459.1961.10482090

[26] Jabbour S, Fouhey D, Kazerooni E, Sjoding MW, Wiens J. Deep Learning Applied to Chest X-Rays: Exploiting and Preventing Shortcuts. In: Doshi-Velez F, Fackler J, Jung K, Kale D, Ranganath R, Wallace B, et al., editors. Proceedings of the 5th Machine Learning for Healthcare Conference. PMLR; 2020. pp. 750–782. Available: https://proceedings.mlr.press/v126/jabbour20a.html.

[27] Heckman J. Varieties of Selection Bias. Am Econ Rev. 1990;80: 313–318.

[28] Kennes, Lieven Nils, Cramer E, Hilgers R, Heussen N. The impact of selection bias on test decisions in randomized clinical trials. Stat Med. 2011;30: 2573–2581. doi: 10.1002/sim.4279 21717489

[29] Datta A, Fredrikson M, Ko G, Mardziel P, Sen S. Proxy Non-Discrimination in Data-Driven Systems. arXiv; 2017. Available: http://arxiv.org/abs/1707.08120.

[30] Prince AER, Schwarcz D. Proxy Discrimination in the Age of Artificial Intelligence and Big Data. Iowa Law Rev. 2019;105: 1257–1318.

[31] Raita Y, Goto T, Faridi MK, Brown DFM, Camargo CA, Hasegawa K. Emergency department triage prediction of clinical outcomes using machine learning models. Crit Care. 2019;23: 64. doi: 10.1186/s13054-019-2351-7 30795786 PMC6387562

[32] Pearl J. Causality: Models, Reasoning, and Inference. 2nd ed. Cambridge University Press; 2009. doi: 10.1017/CBO9780511803161

[33] Yang X, Song Z, King I, Xu Z. A Survey on Deep Semi-Supervised Learning. IEEE Trans Knowl Data Eng. 2023;35: 8934–8954. doi: 10.1109/TKDE.2022.3220219

[34] Wiens J, Creary M, Sjoding MW. AI models in health care are not colour blind and we should not be either. Lancet Digit Health. 2022;4: e399–e400. doi: 10.1016/S2589-7500(22)00092-9 35568691

[35] Movva R, Shanmugam D, Hou K, Pathak P, Guttag J, Garg N, et al. Coarse race data conceals disparities in clinical risk score performance. Proceedings of the 8th Machine Learning for Healthcare Conference. 2023.

[36] LaVeist TA, Pérez-Stable EJ, Richard P, Anderson A, Isaac LA, Santiago R, et al. The Economic Burden of Racial, Ethnic, and Educational Health Inequities in the US. JAMA. 2023;329: 1682. doi: 10.1001/jama.2023.5965 37191700

[37] King G, Nielsen R. Why Propensity Scores Should Not Be Used for Matching. Polit Anal. 2019;27: 435–454. doi: 10.1017/pan.2019.11

[38] Hoffman KM, Trawalter S, Axt JR, Oliver MN. Racial bias in pain assessment and treatment recommendations, and false beliefs about biological differences between blacks and whites. Proc Natl Acad Sci. 2016;113: 4296–4301. doi: 10.1073/pnas.1516047113 27044069 PMC4843483

