# Supplementary figures and images for: Racial differences in laboratory testing as a potential mechanism for bias in AI: A matched cohort analysis in emergency department visits
Source: PLOS Glob Public Health. 2024 Oct 30;4(10):e0003555. doi: 10.1371/journal.pgph.0003555 (PMC11524489; doi:10.1371/journal.pgph.0003555)

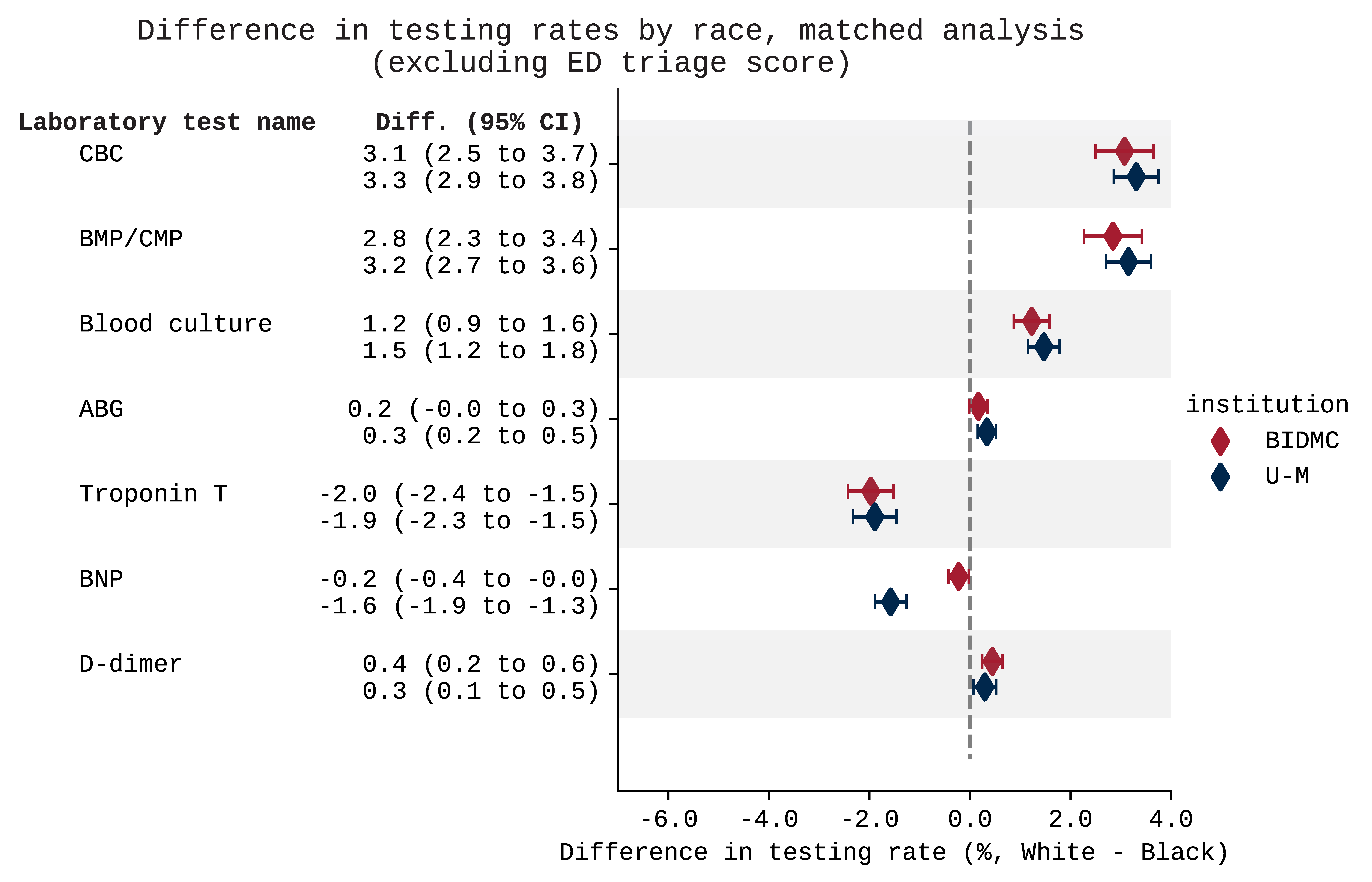

Supplement: S1 Fig — (TIFF) [file pgph.0003555.s001.tiff]

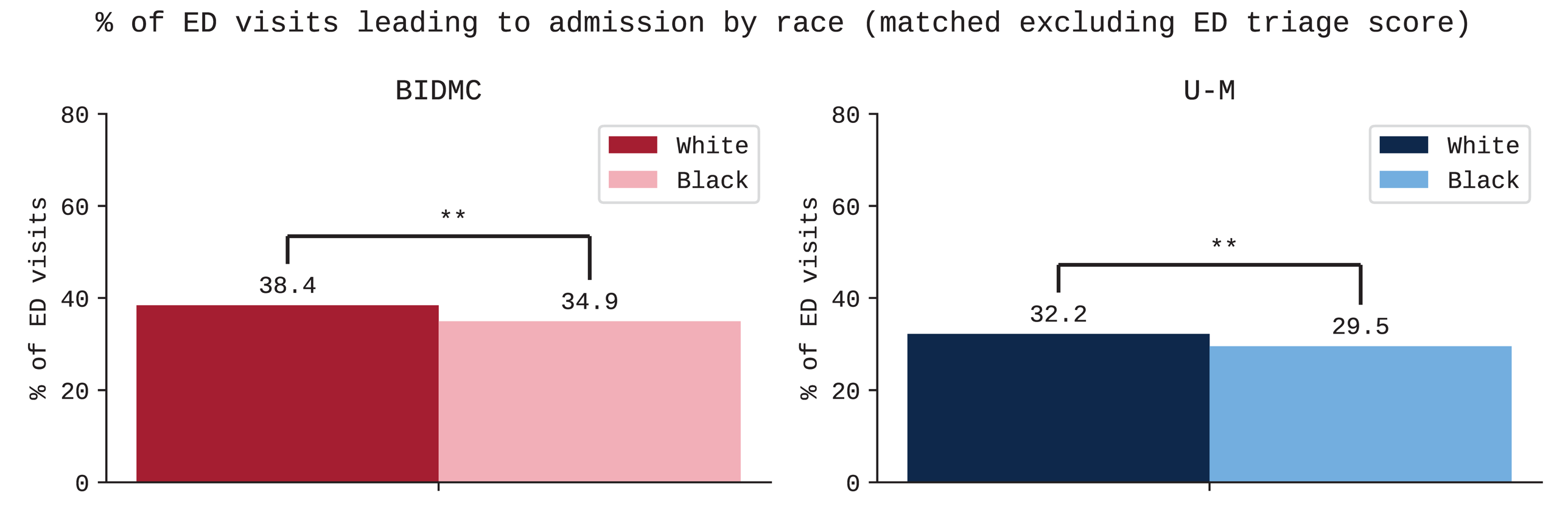

Supplement: S2 Fig — (TIFF) [file pgph.0003555.s002.tiff]

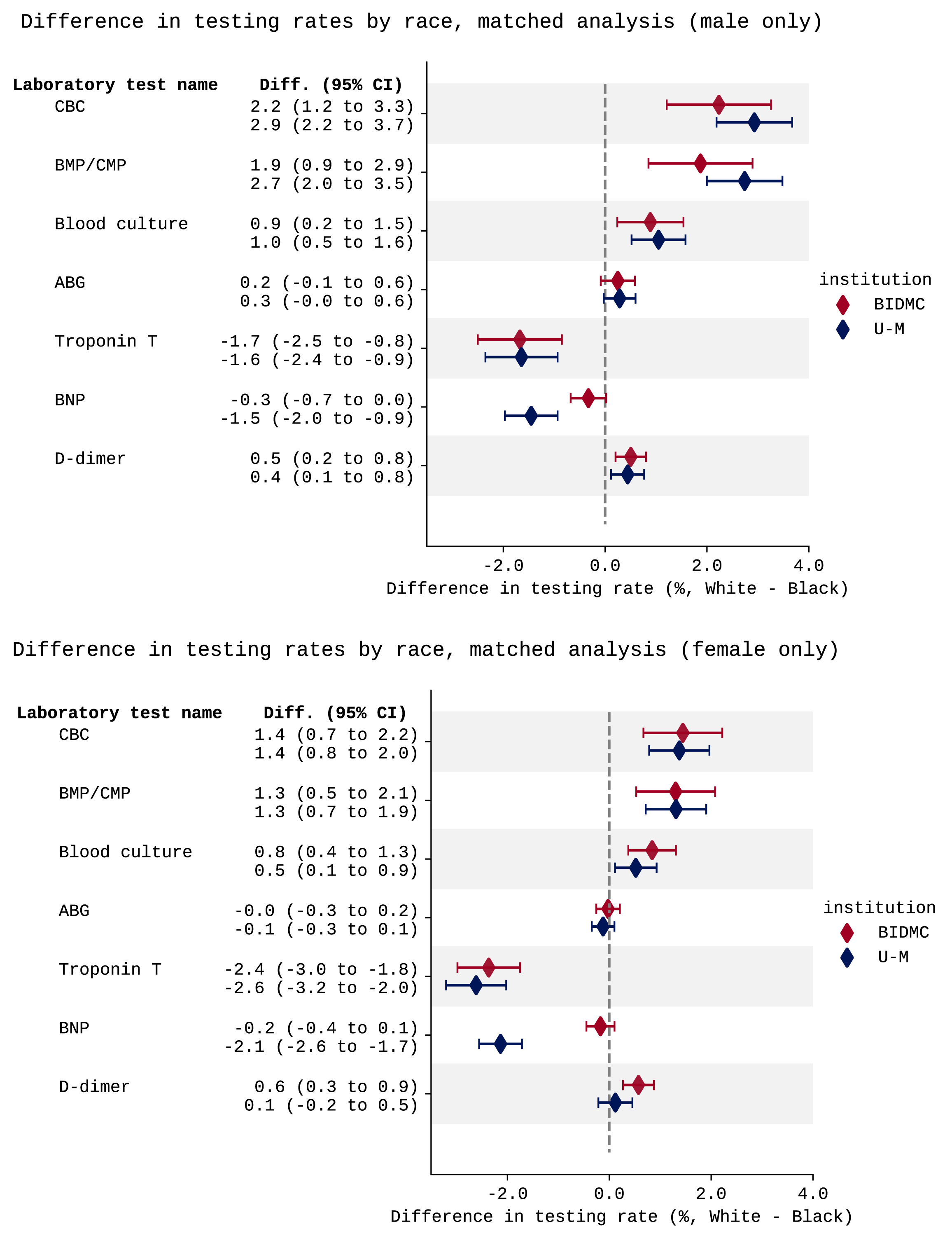

Supplement: S3 Fig — Differences in testing rates between White and Black patients matched on age, biological sex, chief complaint, and ED triage score, stratified by male (top) and female (bottom) patients. (TIFF) [file pgph.0003555.s003.tiff]

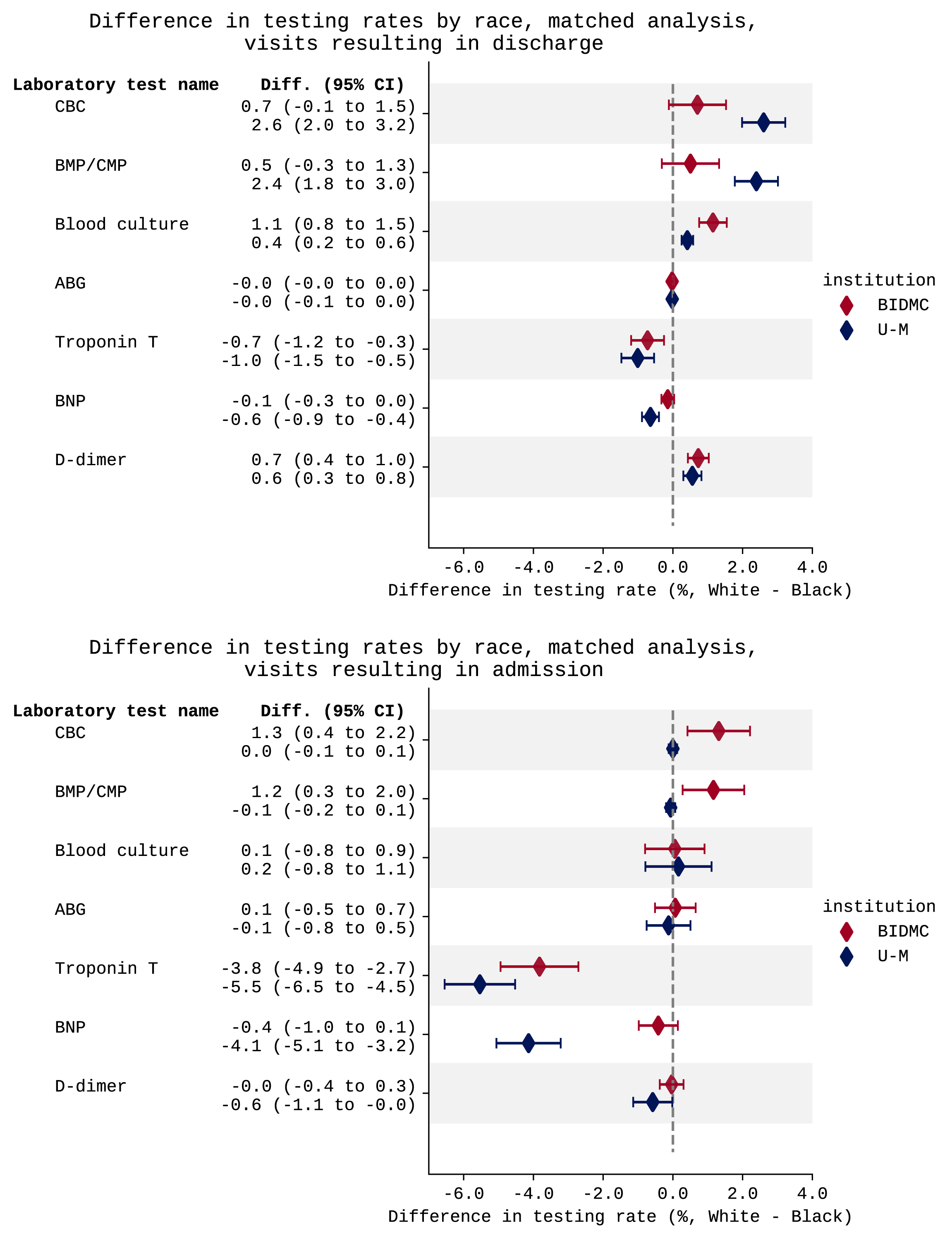

Supplement: S4 Fig — (TIFF) [file pgph.0003555.s004.tiff]
