# Supplementary material for: Racial differences in laboratory testing as a potential mechanism for bias in AI: A matched cohort analysis in emergency department visits
Source: PLOS Glob Public Health. 2024 Oct 30;4(10):e0003555. doi: 10.1371/journal.pgph.0003555 (PMC11524489; doi:10.1371/journal.pgph.0003555)
Supplement: S1 Table — Case-normalized free-text chief complaint for 25 most common chief complaints in the unmatched cohort at BIDMC (left) and U-M (right) among White and Black patients. (PDF) [file pgph.0003555.s005.pdf]

BIDMC			U-M		
**Complaint text[a]**	**White (n=244,387)**	**Black (n=92,437)**	**Complaint text**	**White (n=439,005)**	**Black (n=102,269)**
abd pain	9,566 (3.9)	3,565 (3.9)	chest pain	22,140 (5.0)	5,825 (5.7)
chest pain	7,923 (3.2)	3,812 (4.1)	shortness of breath	18,186 (4.1)	4,025 (3.9)
s/p fall	5,597 (2.3)	1,077 (1.2)	abdominal pain - generalized	15,173 (3.5)	3,532 (3.5)
dyspnea	4,698 (1.9)	1,899 (2.1)	back pain	8,643 (2.0)	2,102 (2.1)
wound eval	3,217 (1.3)	1,135 (1.2)	fall	8,590 (2.0)	9,36 (0.9)
back pain	2,726 (1.1)	1,282 (1.4)	alcohol intoxication	6,913 (1.6)	2,482 (2.4)
headache	2,566 (1.1)	1,494 (1.6)	altered mental status	7,212 (1.6)	1,194 (1.2)
fever	2,546 (1.0)	467 (0.5)	wound check	6,860 (1.6)	1,457 (1.4)
weakness	2,045 (0.8)	620 (0.7)	abnormal lab	6,673 (1.5)	1,409 (1.4)
syncope	1,934 (0.8)	509 (0.6)	flank pain	6,975 (1.6)	995 (1.0)
altered mental status	1,793 (0.7)	557 (0.6)	vomiting	6,066 (1.4)	1,661 (1.6)
dizziness	1,564 (0.6)	676 (0.7)	headache	5,974 (1.4)	1,736 (1.7)
n/v	1,514 (0.6)	587 (0.6)	leg pain	4,817 (1.1)	1,433 (1.4)
etoh	1,508 (0.6)	541 (0.6)	fever	5,382 (1.2)	774 (0.8)
abnormal labs	1,505 (0.6)	540 (0.6)	dizziness	4,884 (1.1)	1,119 (1.1)
brbpr	1,502 (0.6)	521 (0.6)	fatigue	4,525 (1.0)	989 (1.0)
seizure	1,470 (0.6)	521 (0.6)	eye problem	4,266 (1.0)	767 (0.7)
lower back pain	1,347 (0.6)	994 (1.1)	motor vehicle crash	3,577 (0.8)	994 (1.0)
mvc	1,305 (0.5)	972 (1.1)	syncope	3789 (0.9)	686 (0.7)
palpitations	1,241 (0.5)	440 (0.5)	seizures	3317 (0.8)	891 (0.9)
abdominal pain	1,164 (0.5)	427 (0.5)	abdominal pain - lower	3,277 (0.7)	903 (0.9)
abd pain n/v	1,161 (0.5)	608 (0.7)	abdominal pain - right lower quadrant	3,526 (0.8)	471 (0.5)

BIDMC (continued)			U-M (continued)		
**Complaint text (continued)**	**White (n=244,387; continued)**	**Black (n=92,437; continued)**	**Complaint text (continued)**	**White (n=439,005; continued)**	**Black (n=102,269; continued)**
chest pain dyspnea	1,077 (0.4)	563 (0.6)	cough	2,556 (0.6)	949 (0.9)
sore throat	996 (0.4)	681 (0.7)	postop problem	2,999 (0.7)	462 (0.5)
rash	961 (0.4)	654 (0.7)	palpitations	3,015 (0.7)	435 (0.4)

[a]Word sorting omitted for readability (there were no complaints with duplicate words in the 25 most common complaints).
