# Supplementary material for: Racial differences in laboratory testing as a potential mechanism for bias in AI: A matched cohort analysis in emergency department visits
Source: PLOS Glob Public Health. 2024 Oct 30;4(10):e0003555. doi: 10.1371/journal.pgph.0003555 (PMC11524489; doi:10.1371/journal.pgph.0003555)
Supplement: S2 Table — (PDF) [file pgph.0003555.s006.pdf]

**Institution**	**BIDMC**			**U-M**		
**Race**	**White (n=244,387)**	**Black (n=92,437)**	***P* value**	**White (n=439,005)**	**Black (n=102,269)**	***P* value**
Complete blood count	170,081 (69.6)	55,890 (60.5)	<.001	335,263 (76.4)	72,078 (70.5)	<.001
Metabolic panel	170,482 (69.8)	56,198 (60.8)	<.001	334,946 (76.3)	71,990 (70.4)	<.001
Blood culture	34,024 (13.9)	9,273 (10.0)	<.001	65,038 (14.8)	11,083 (10.8)	<.001
Arterial blood gas	10,248 (4.2)	2,297 (2.5)	<.001	19,939 (4.5)	3,318 (3.2)	<.001
Troponin T	46,018 (18.8)	16,843 (18.2)	<.001	117,086 (26.7)	25,820 (25.3)	<.001
Brain natriuretic peptide	9,105 (3.7)	2,900 (3.1)	<.001	54,645 (12.5)	11,694 (11.4)	<.001
D-dimer	5,148 (2.1)	2,411 (2.6)	<.001	21,332 (4.9)	5,090 (5.0)	.12
