# Supplementary material for: Racial differences in laboratory testing as a potential mechanism for bias in AI: A matched cohort analysis in emergency department visits
Source: PLOS Glob Public Health. 2024 Oct 30;4(10):e0003555. doi: 10.1371/journal.pgph.0003555 (PMC11524489; doi:10.1371/journal.pgph.0003555)
Supplement: S3 Table — (PDF) [file pgph.0003555.s007.pdf]

Institution	BIDMC			U-M		
**Race**	**White (n=47,160)**	**Black (n=47,160)**	***P* value**	**White (n=70,755)**	**Black (n=70,755)**	***P* value**
Median age, years (IQR)	46 (30 - 61)	46 (30 - 61)	N/A	44 (30 - 59)	44 (30 - 59)	N/A
Sex (% Female)	29,481 (62.5)	29,481 (62.5)	N/A	40,929 (57.8)	40,929 (57.8)	N/A
ED triage score (%)[a]						
1	1,342 (2.9)	1,342 (2.9)	N/A	435 (0.6)	435 (0.6)	N/A
2	11,800 (25.0)	11,800 (25.0)		24,785 (35.0)	24,785 (35.0)	
3	30,397 (64.5)	30,397 (64.5)		36,352 (51.4)	36,352 (51.4)	
4	3,579 (7.6)	3,579 (7.6)		8,765 (12.4)	8,765 (12.4)	
5	42 (0.1)	42 (0.1)		418 (0.6)	418 (0.6)	
Mean ED triage score (SD)	2.77 (0.63)	2.77 (0.63)	N/A	2.77 (0.69)	2.77 (0.69)	N/A
Admitted to hospital (%)	17,775 (37.7)	16,523 (35.0)	<.001	22,172 (31.3)	21,072 (29.8)	<.001

[a]Percentages may not sum to 100.0% due to rounding. Null or missing ED triage scores are removed prior to matching.
