# Supplementary material for: Racial differences in laboratory testing as a potential mechanism for bias in AI: A matched cohort analysis in emergency department visits
Source: PLOS Glob Public Health. 2024 Oct 30;4(10):e0003555. doi: 10.1371/journal.pgph.0003555 (PMC11524489; doi:10.1371/journal.pgph.0003555)
Supplement: S4 Table — (PDF) [file pgph.0003555.s008.pdf]

**Institution**	**BIDMC**			**U-M**		
**Race**	**White (n=47,160)**	**Black (n=47,160)**	***P* value**	**White (n=70,755)**	**Black (n=70,755)**	***P* value**
Complete blood count	29,724 (63.0)	28,903 (61.3)	<.001	52,072 (73.6)	50,635 (71.6)	<.001
Metabolic panel	29,823 (63.2)	29,108 (61.7)	<.001	51,914 (73.4)	50,561 (71.5)	<.001
Blood culture	4,870 (10.3)	4,465 (9.5)	<.001	8,019 (11.3)	7,493 (10.6)	<.001
Arterial blood gas	1,103 (2.3)	1,066 (2.3)	.42	2,298 (3.3)	2,263 (3.2)	.61
Troponin T	8,119 (17.2)	9,113 (19.3)	<.001	17,186 (24.3)	18,746 (26.5)	<.001
Brain natriuretic peptide	1,342 (2.8)	1,451 (3.1)	.04	7,174 (10.1)	8,481 (12.0)	<.001
D-dimer	1,573 (3.3)	1,315 (2.8)	<.001	3,965 (5.6)	3,784 (5.4)	.04
