# Supplementary material for: Racial differences in laboratory testing as a potential mechanism for bias in AI: A matched cohort analysis in emergency department visits
Source: PLOS Glob Public Health. 2024 Oct 30;4(10):e0003555. doi: 10.1371/journal.pgph.0003555 (PMC11524489; doi:10.1371/journal.pgph.0003555)
Supplement: S5 Table — (PDF) [file pgph.0003555.s009.pdf]

**Institution**	**BIDMC**			**U-M**		
**Race**	**White (n=54,744)**	**Black (n=54,744)**	***P* value**	**White (n=77,417)**	**Black (n=77,417)**	***P* value**
Complete blood count	34,598 (63.2)	32,915 (60.1)	<.001	57,204 (73.9)	54,643 (70.6)	<.001
Metabolic panel	34,699 (63.4)	33,142 (60.5)	<.001	57,023 (73.7)	54,582 (70.5)	<.001
Blood culture	5,845 (10.7)	5,173 (9.4)	<.001	9,330 (12.1)	8,193 (10.6)	<.001
Arterial blood gas	1,368 (2.5)	1,277 (2.3)	.07	2,813 (3.6)	2,553 (3.3)	<.001
Troponin T	9,243 (16.9)	10,323 (18.9)	<.001	18,585 (24.0)	20,051 (25.9)	<.001
Brain natriuretic peptide	1,554 (2.8)	1,676 (3.1)	.03	7,866 (10.2)	9,089 (11.7)	<.001
D-dimer	1,720 (3.1)	1,478 (2.7)	<.001	4,281 (5.5)	4,053 (5.2)	.01
