# Supplementary material for: Racial differences in laboratory testing as a potential mechanism for bias in AI: A matched cohort analysis in emergency department visits
Source: PLOS Glob Public Health. 2024 Oct 30;4(10):e0003555. doi: 10.1371/journal.pgph.0003555 (PMC11524489; doi:10.1371/journal.pgph.0003555)
Supplement: S6 Table — (PDF) [file pgph.0003555.s010.pdf]

Institution	BIDMC						U-M					
**Biological sex**	**Female**			**Male**			**Female**			**Male**		
**Race**	**White (n=29, 481)**	**Black (n=29, 481)**	***P* value**	**White (n=17,6 79)**	**Black (n=17,6 79)**	***P* value**	**White (n=40,9 29)**	**Black (n=40,9 29)**	***P* value**	**White (n=29,8 26)**	**Black (n=29,8 26)**	***P* value**
Complete blood count	19,086 (64.7)	18,660 (63.3)	<.001	10,638 (60.2)	10,243 (57.9)	<.001	31,092 (76.0)	30,529 (74.6)	<.001	20,980 (70.3)	20,106 (67.4)	<.001
Metabolic panel	19,076 (64.7)	18,692 (63.4)	<.001	10,747 (60.8)	10,416 (58.9)	<.001	30,909 (75.5)	30,373 (74.2)	<.001	21,005 (70.4)	20,188 (67.7)	<.001
Blood culture	2,860 (9.7)	2,612 (8.9)	<.001	2,010 (11.4)	1,853 (10.5)	.007	4,144 (10.1)	3,931 (9.6)	.01	3,875 (13.0)	3,562 (11.9)	<.001
Arterial blood gas	612 (2.1)	619 (2.1)	.84	491 (2.8)	447 (2.5)	.15	1,091 (2.7)	1,141 (2.8)	.29	1,207 (4.1)	1,122 (3.8)	.08
Troponin T	4,826 (16.4)	5,524 (18.7)	<.001	3,293 (18.6)	3,589 (20.3)	<.001	9,521 (23.3)	10,591 (25.9)	<.001	7,665 (25.7)	8,155 (27.3)	<.001
Brain natriuretic peptide	860 (2.9)	911 (3.1)	.22	482 (2.7)	540 (3.1)	.07	3,880 (9.5)	4,754 (11.6)	<.001	3,294 (11.0)	3,727 (12.5)	<.001
D-dimer	1,158 (3.9)	989 (3.4)	<.001	415 (2.3)	326 (1.8)	<.001	2,627 (6.4)	2,578 (6.3)	.49	1,338 (4.5)	1,206 (4.0)	.008
