# Supplementary material for: Racial differences in laboratory testing as a potential mechanism for bias in AI: A matched cohort analysis in emergency department visits
Source: PLOS Glob Public Health. 2024 Oct 30;4(10):e0003555. doi: 10.1371/journal.pgph.0003555 (PMC11524489; doi:10.1371/journal.pgph.0003555)
Supplement: S7 Table — (PDF) [file pgph.0003555.s011.pdf]

**Institution**	**BIDMC**			**U-M**		
**Race**	**White (n=28,531)**	**Black (n=28,531)**	***P* value**	**White (n=47,940)**	**Black (n=47,940)**	***P* value**
Complete blood count	14,515 (50.9)	14,314 (50.2)	.09	29,623 (61.8)	28,375 (59.2)	<.001
Metabolic panel	14,543 (51.0)	14,399 (50.5)	.23	29,456 (61.4)	28,308 (59.1)	<.001
Blood culture	1,945 (6.8)	1,617 (5.7)	<.001	945 (2.0)	746 (1.6)	<.001
Arterial blood gas	5 (0.0)	11 (0.0)	.13	62 (0.1)	71 (0.2)	.49
Troponin T	2,493 (8.7)	2,700 (9.5)	.003	7,612 (15.9)	8,095 (16.9)	<.001
Brain natriuretic peptide	337 (1.2)	379 (1.3)	.11	1,692 (3.5)	2,000 (4.2)	<.001
D-dimer	1,090 (3.8)	882 (3.1)	<.001	2,224 (4.6)	1,956 (4.1)	<.001
