# Supplementary material for: Racial differences in laboratory testing as a potential mechanism for bias in AI: A matched cohort analysis in emergency department visits
Source: PLOS Glob Public Health. 2024 Oct 30;4(10):e0003555. doi: 10.1371/journal.pgph.0003555 (PMC11524489; doi:10.1371/journal.pgph.0003555)
Supplement: S8 Table — (PDF) [file pgph.0003555.s012.pdf]

**Institution**	**BIDMC**			**U-M**		
**Race**	**White (n=14,355)**	**Black (n=14,355)**	***P* value**	**White (n=18,684)**	**Black (n=18,684)**	***P* value**
Complete blood count	11,802 (82.2)	11,613 (80.9)	.004	18,617 (99.6)	18,617 (99.6)	1
Metabolic panel	11,894 (82.9)	11,727 (81.7)	.009	18,599 (99.6)	18,611 (99.6)	.38
Blood culture	2,334 (16.3)	2,326 (16.2)	.90	6,063 (32.5)	6,033 (32.3)	.75
Arterial blood gas	979 (6.8)	969 (6.8)	.81	1,996 (10.7)	2,019 (10.8)	.71
Troponin T	5,041 (35.1)	5,590 (38.9)	<.001	8,358 (44.7)	9,392 (50.3)	<.001
Brain natriuretic peptide	871 (6.1)	931 (6.5)	.14	5,093 (27.3)	5,866 (31.4)	<.001
D-dimer	314 (2.2)	319 (2.2)	.84	1,497 (8.0)	1,605 (8.6)	.04
