# Supplementary material for: Racial differences in laboratory testing as a potential mechanism for bias in AI: A matched cohort analysis in emergency department visits
Source: PLOS Glob Public Health. 2024 Oct 30;4(10):e0003555. doi: 10.1371/journal.pgph.0003555 (PMC11524489; doi:10.1371/journal.pgph.0003555)
Supplement: S1 Appendix — (DOCX) [file pgph.0003555.s013.docx]

METHODS SUPPLEMENT

**Laboratory test extraction**

In MIMIC-IV, each laboratory value was stored in a separate record. For laboratory tests with a single associated value (*e.g.,* BNP, Troponin T), the admission identifier associated with all lab events containing the relevant laboratory value were collected to determine admissions in which the relevant laboratory test was ordered. For hospital visits without an admission identifier (*i.e.,* ED visits that did not result in admission), the chart time of the relevant lab value was cross-referenced with the start and end times of the visit, from which an ED visit identifier was associated with each relevant laboratory test. At BIDMC, laboratory tests with multiple associated values (*e.g.,* CBC, metabolic panels), all relevant laboratory values for each test were collected and grouped by chart time, then cross-referenced with an admission identifier or ED visit identifier as described above. Laboratory test records were constructed similarly in the U-M data.

**Additional data processing and matching details**

For BIDMC, age information needed to be reconstructed from their shifted admission dates, since MIMIC-IV shifts timestamps for each patient for de-identification. To perform exact matching, visits with unknown or miscellaneous complaints were excluded from the matched analysis, as were visits with unknown or missing ED triage score.

RESULTS SUPPLEMENT

**Sensitivity Analysis (Alternative Matching Strategy)**

Since ED triage score is potentially a collider with respect to race and other confounders, we run experiments with an alternate matching strategy, performing 1:1 exact matching on race, biological sex, and chief complaint (*i.e.,* excluding ED triage score with respect to the main paper). This yields a population of 109,656 and 154,384 ED visits at BIDMC and U-M, respectively. This represents 59.3% of Black patients and 22.4% of White patients at BIDMC, and 75.5% of Black patients and 17.6% of White patients at U-M.

Overall trends were similar to those identified in the primary analysis. However, Black patients were significantly less likely than White patients to receive a blood culture (BIDMC: 10.7% vs. 9.4%, difference: 1.2%, 95% CI: 0.9% to 1.6%, p<.001) at both institutions (instead of U-M only). Trends are similar at U-M (S1 Fig). We note that, under this matching strategy, White patients remained significantly more likely to be admitted following the ED visit at both institutions (BIDMC: 38.4% vs. 34.9%, difference: 3.5%, 95% CI: 2.9% to 4.1%, p<.001). Trends in hospital admission rates are similar at U-M (S2 Fig). Full results are reported in S1 Fig and S4 Table.

**Intersectional Analysis**

Black male patients were significantly less likely than White male patients to receive a CBC (BIDMC: 60.2% vs. 57.9%, difference 2.3%, 95% CI: 1.2% to 3.3%, p<.001) or metabolic panel (BIDMC: 60.8% vs. 58.9%, difference 1.9%, 95% CI: 0.9% to 2.9%, p<.001). Similarly, White patients were significantly less likely than Black patients to receive a troponin (BIDMC: 18.6% vs. 20.3%, difference -1.7%, 95% CI: -2.5% to -0.8%, p<.001) test. Trends are similar in female patients (S3 Fig, bottom) and at U-M (S5 Fig). In contrast to the primary analysis, after disaggregating by biological sex, there was no significant difference in blood culture or BNP rates across race in both male and female patients. Note that we use a Bonferroni correction factor of 28 for this disaggregated analysis. Full results are reported in S3 Fig and S5 Table.

**Subgroup analysis with respect to admission status**

In the subgroup analysis of visits resulting in discharge, matching on age, sex, ED triage score, and chief complaint yielded a population of 57,062 and 95,880 ED visits at BIDMC and U-M, respectively. At BIDMC, this matched subgroup included 48.2% and 23.8% of all visits resulting in discharge by Black and White patients, respectively. At U-M, this matched subgroup included 66.8% and 17.6% of visits resulting in discharge by Black and White patients, respectively. Within such visits, White patients were significantly more likely than Black patients to receive a blood culture (BIDMC: 6.8% vs. 5.7%, difference 1.1%, 95% CI: 0.8% to 1.5%, p<.001) or *d-*dimer (BIDMC: 3.8% vs. 3.1%, difference 0.7%, 95% CI: 0.4% to 1.0%, p<.001). Trends were similar at U-M (S4 Fig, top). See S7 Table for the testing rates shown in S4 Fig (top).

Among patients admitted to the hospital, matching on age, sex, ED triage score, and chief complaint yielded a population of 28,710 and 37,368 ED visits at BIDMC and U-M, respectively. At both institutions (S4 Fig, bottom), White patients were significantly less likely than Black patients to receive a troponin test (BIDMC: 35.1% vs. 38.9%, difference -3.8%, 95% CI: -5.8% to -1.9%, p<.001). The remaining testing differences in this subgroup were not statistically significant at both institutions. See S8 Table for the testing rates shown in S4 Fig (bottom).
